# Investigating the Biocontrol and Plant Growth-Promoting Potential of *Pseudomonas yamanorum* for Sustainable Management of Tomato Early Blight (*Alternaria alternata*)

**DOI:** 10.3390/plants14203117

**Published:** 2025-10-10

**Authors:** Lobna Hajji-Hedfi, Takwa Wannassi, Amira Khlif, Nyasha J. Kavhiza, Nazih Y. Rebouh

**Affiliations:** 1 Regional Centre of Agricultural Research of Sidi Bouzid, Gafsa Road Km 6, B.P. 357, Sidi Bouzid 9100, Tunisia; wntakwa1@gmail.com (T.W.);; 2 Laboratory of Agriculture Production Systems and Sustainable Development (LR03AGR02), Department of Agricultural Production, Higher School of Agriculture of Mograne, University of Carthage, Mograne Zaghouan, Tunis 1121, Tunisia; 3 Department of Environmental Management, Institute of Environmental Engineering, RUDN University, 6 Miklukho-Maklaya St., 117198 Moscow, Russia; njkavhiza@rocketmail.com

**Keywords:** *Alternaria alternata*, antifungal activity, biocontrol, plant-growth promoting, *Pseudomonas yamanorum*, salicylic acid, tomato

## Abstract

Tomato (*Solanum lycopersicum* L.) is among the most economically significant and nutritionally valuable vegetable crops grown globally. However, fungal diseases such as Early Blight caused by *Alternaria alternata* are a major factor limiting yield and fruit quality in tomato production. This study investigates the biocontrol potential of locally isolated rhizobacterium *Pseudomonas yamanorum* against *A. alternata*, the causal agent of early blight in tomato, under both in vitro and in planta conditions. In vitro assays demonstrated significant antifungal activity; in the dual confrontation assay, *P. yamanorum* (10^8^ CFU/mL) reduced *A. alternata* mycelial growth by 68.7%, while spore germination was inhibited by 88.7%. In planta trials demonstrated that plants treated with *P. yamanorum* (10^7^ CFU/mL) alone exhibited the lowest disease severity (2.5). The treatments also significantly enhanced plant growth, with shoot length reaching 45 cm versus 26 cm in infected controls. Biochemical analyses revealed increased catalase (94.84 units mg^−1^ protein min^−1^), peroxidase (5.83), and ascorbate peroxidase (67.01) activities in treated plants. Total polyphenol and protein contents also increased (0.81 mg/g and 15.82 mg/g, respectively). Furthermore, *P. yamanorum* treatments maintained fruit quality parameters such as firmness (3.13), sugar content (6.43 °Brix), and juice yield (55.88%), while reducing malondialdehyde (2.02 µmol/g Dry Weight) and electrical conductivity (0.59 mS/cm). These findings highlight *P. yamanorum* as a promising biocontrol agent and plant growth-promoting bacteria that improve disease resistance, which can be combined with salicylic acid to further enhance crop vigor and fruit quality under biotic stress.

## 1. Introduction

The tomato (*Solanum lycopersicum* L.) is among the most economically and nutritionally important vegetable crops cultivated worldwide. This vegetable is widely consumed and valued for its rich content of health-promoting compounds, including lycopene, other antioxidants, phytochemicals, and essential nutrients such as iron, potassium, vitamin C, and folate [1].

Global tomato production is on an upward trajectory, with an estimated 192 million tonnes harvested in 2023. China leads as the largest producer, followed by India, Turkey, and the United States of America [2,3]. Tomato cultivation is an important enterprise in the Mediterranean countries, where Tunisia is one of the principal producers, with an annual production of approximately 1.3 million tonnes [3]. These levels of production therefore underscore the crop’s critical role in both regional and global food systems.

Despite its agronomic and economic importance, tomato cultivation is bedeviled by substantial challenges emanating from biotic stresses, particularly fungal diseases. One of the most destructive tomato diseases is early blight, caused by *Alternaria species* such as *A. solani* and *A. alternata* [4]. Early blight is characterized by concentric necrotic lesions on the leaves, stem, and fruit, leading to premature defoliation, thus reduced photosynthetic activity and consequent decrease in yield and quality [5]. This disease is particularly severe in warm and humid climates, typical of Mediterranean and subtropical regions, where yield losses range from 35% to 80% [4]. Prolonged durations of high humidity as well as temperatures exacerbate the incidence and severity of the disease [6].

In addition to quantitative losses, early blight also adversely affects fruit quality, including potential contamination with mycotoxins as well as diminished nutritional value [7].

To offset this challenge, Tunisian farmers have increasingly relied on chemical methods of control to sustain agricultural productivity in recent decades. The Tunisian agricultural sector has an annual pesticide consumption equating to 3299.07 metric tonnes, approximately, ranking 89th globally. Moreover, the pesticide consumption is projected to rise to 4160 tonnes by 2026, a 2.1% annual increase from 3640 tonnes in 2021 [8].

Synthetic fungicides are extensively applied to control fungal pathogens in tomato cultivation. Nonetheless, their intensive and often indiscriminate use has raised serious environmental, ecological, and public health concerns. Excessive application disrupts soil microbial diversity, as well as contributes to surface and groundwater contamination, posing serious potential human health risks [9,10]. Furthermore, persistent compounds like copper-based fungicides, including formulations like cupric hydroxide combined with ethylene bisdithiocarbamate, widely used in tomato production systems, aggravate the environmental issues associated with this enterprise. These compounds accumulate in soils, posing considerable risk to non-target organisms, including pollinators (e.g., bees), soil invertebrates (e.g., earthworms), and aquatic ecosystems [11]. In addition, frequent fungicide application exerts selective pressure on pathogen populations, leading to the development of resistant strains, limiting the long-term efficacy of chemical disease control measures [12].

As a responsive action, recent research has increasingly focused on the development and application of biological control agents (BCAs) against tomato pathogens worldwide. Among the most extensively studied organisms are beneficial rhizobacteria, such as *Pseudomonas* spp. and *Bacillus* spp., as well as fungal genera such as Trichoderma [13].

Similarly, bacterial isolates from the genus Bacillus have emerged as promising BCAs for the management of tomato diseases [14]. Bacillus-based biopesticides not only suppress pathogens and promote plant health but also serve as a solution to diminish environmental degradation in agriculture. These traits make them highly valued for their eco-friendliness as well as compatibility with organic farming systems under various cultivation conditions [14,15].

Among BCAs, *Pseudomonas* spp. are particularly recognized as plant growth-promoting rhizobacteria (PGPR) with considerable antifungal properties [16]. Numerous studies have illustrated that the tomato rhizosphere is frequently colonized by *Pseudomonas fluorescens*, conferring multiple benefits to the host plant, for instance, increased biomass, enhanced nutrient uptake, and regulation of plant hormone balance [17,18]. It is also important to note that *Pseudomonas* spp. are well known for inducing systemic resistance (ISR) in plants. This defense response is largely mediated by microbial-derived lipopeptides, through the stimulation of the plant immune system by activation of the jasmonic acid and ethylene signaling pathways [19].

In practical terms, within the Mediterranean context, the use of BCAs as alternative options to chemical fungicides has been successfully demonstrated in Tunisia. Recent efforts have shifted towards the identification of locally adapted BCAs; notably, a strain of *P. yamanorum* was isolated from the tomato fields and shown to exhibit strong antifungal activity against grapevine pathogens [20]. A field trial carried out in Tunisia further consolidated that a microbial consortium composed of *P. yamanorum* and *Trichoderma longibrachiatum* significantly reduced the severity of fungal diseases in grapevines by enhancing plant resistance. These findings are indicative of the potential vested in locally sourced microbial consortia for effective and sustainable disease management in Mediterranean farming systems.

These previously obtained results illustrate that *P. yamanorum* is a well-adapted BCA for warm, semi-arid agroecosystems. Furthermore, beyond the application of biocontrol inoculants alone, there is a developing interest in combining BCAs with plant defense elicitors, such as salicylic acid (SA), an important signaling hormone that activates systemic acquired resistance in tomato [20,21].

Investigating *P. yamanorum* in combination with SA treatments attempts to fill a large research gap in the Mediterranean context. This modus operandi leverages plant defense signaling and locally sourced beneficial bacteria to sustainably manage early blight disease. Therefore, the present study was designed to evaluate the antagonistic and antifungal potential of *P. yamanorum* alone and in combination with SA against *A. alternata*, in a bid to control early blight in tomato. Moreover, the study assessed the efficacy of these treatments in reducing disease severity and enhancing plant health under greenhouse conditions.

## 2. Results

### 2.1. Physiological and Biochemical Characterization of Pseudomonas yamanorum

**Figure 1:**
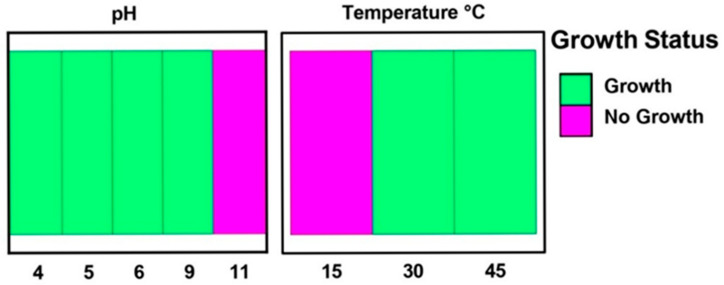
pH and temperature fitness profile of *Pseudomonas yamanorum* on LB medium.

To understand how environmental pH affected *P. yamanorum*’s viability, the strain was cultivated on LB medium with a range of pH values, from acidic (pH 4) to extremely alkaline (pH 11). When exposed to slightly acidic to moderately basic conditions (pH 4 to 9), *P. yamanorum* showed vigorous growth. The fact that no growth was observed at pH 11 illustrates that extremely alkaline environments prevent the growth of the bacteria. Based on these results, it appears that *P. yamanorum* can adapt to a wide pH range, with near-neutral to slightly alkaline environments promoting optimal growth (Figure 1). Furthermore, *P. yamanorum* showed a considerable degree of thermotolerance by positively growing at 30 °C, as well as at 45 °C. However, its metabolic activity appears to be limited at lower temperatures, as no growth was observed below 15 °C. According to these results, *P. yamanorum* is well adapted to mesophilic and moderately high-temperature environments and less appropriate for colder climates (Figure 1).

### 2.2. Extracellular Enzymatic Activity and Plant-Growth-Promoting Traits of Pseudomonas yamanorum

**Figure 2:**
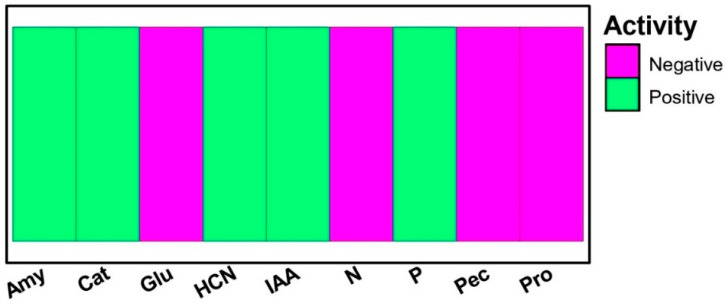
Biochemical profile of *Pseudomonas yamanorum* for plant growth-promoting traits and extracellular enzymatic activity. (Amy: Amylase, Cat: Catalase, Glu: Glucanase, HCN: Hydrogen cyanide, IAA: Indole-3-acetic acid, N: Nitrogen fixation, P: Phosphate solubilization, Pec: Pectinase, Pro: protease). Green bars indicate positive activity; pink bars indicate negative activity.

Trials conducted showed that the bacterial strain used in this study is capable of decomposing hydrogen peroxide due to the presence of catalase. Furthermore, the bacterium exhibits amylolytic activity, as evidenced by the appearance of a clear halo around the colony, indicating starch hydrolysis. The production of antibiotics in *P. yamanorum* was indicated by an orange-red color, the intensity of which varied depending on the amount of HCN produced. As for the bio-stimulant potential through the PGP traits, the bacterial strain showed a positive reaction with the Salkowski reagent, indicated by the appearance of a pink color, which reflects the presence of IAA. Moreover, the results also showed that the bacterium solubilized phosphate (Figure 2).

### 2.3. In Vitro, Antifungal Activities of Pseudomonas yamaranoum Against Alternaria alternata

**Figure 3:**
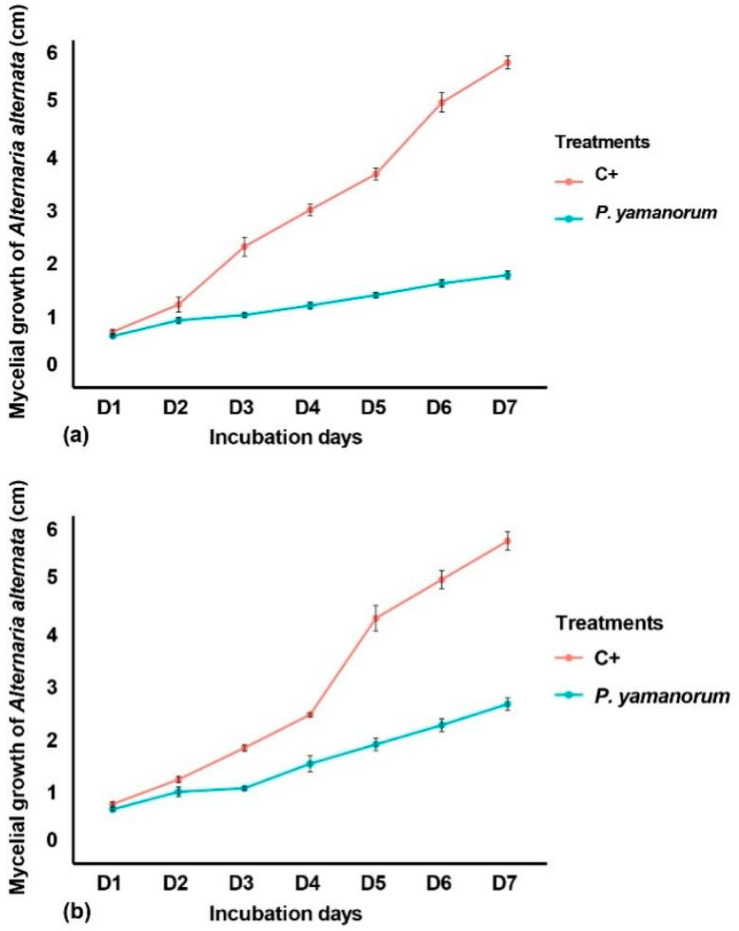
Temporal dynamics of *Alternaria alternata* mycelial growth in seven days under confrontation with *Pseudomonas yamanorum.* (**a**): Direct dual culture assay, (**b**): Indirect confrontation assay (C+: Positive control, *Alternaria alternata* only). The value represents the mean of the 10 replicates ± standard error.

The in vitro trials demonstrated that *P. yamanorum* possesses an important antagonistic impact at a standard concentration of 10^8^ CFU mL^−1^ against *A. alternata* in both direct (Figure 3a) and indirect (Figure 3b) confrontation tests.

In the dual culture assay, visible inhibition of mycelial growth was observed over the 7-day incubation period. The radial growth of *A. alternata* in the control plates reached 5.9 cm, while in the presence of *P. yamanorum*, fungal growth was significantly reduced to 1.84 cm, corresponding to a mean inhibition percentage of 68.70% (Figure 3a). In the indirect confrontation assay, the bacterial strain and the fungal pathogen were physically separated; a notable inhibition of *A. alternata* mycelial growth was observed, consistent with the results obtained in the direct confrontation assay. After the 7-day incubation period, fungal colonies reached an average of 2.73 cm, versus 5.83 cm in control plates, with a calculated inhibition rate of 46.86% (Figure 3b). These results indicate that *P. yamanorum* produces antifungal compounds that are both volatile and diffusible, which may inhibit the growth of *A. alternata* in vitro.

Three concentrations, 10% (C1), 20% (C2), and 40% (C3), were employed to assess the antifungal activity of *P. yamanorum* culture filtrate, i.e., *A. alternata* mycelial growth inhibition (MGI%) and growth rate (MGR) retardation. Significant (*p* < 0.01) variation was noted amongst the tested concentrations.

**Figure 4:**
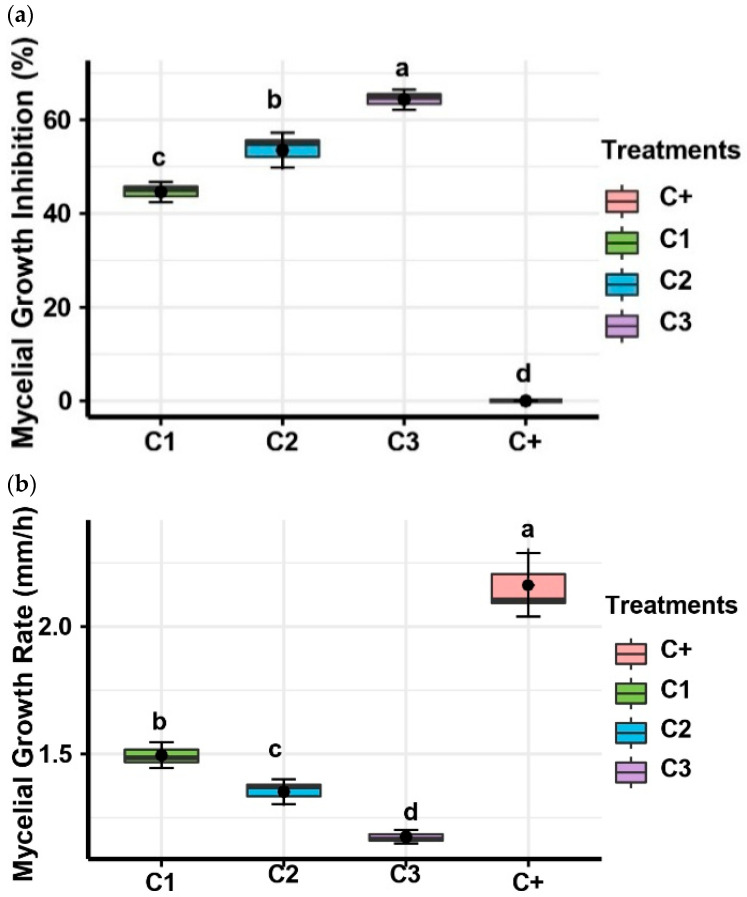
Effect of *Pseudomonas yamanorum* filtrates at different concentrations (10% (C1), 20% (C2), and 40% (C3)) on the mycelial growth inhibition (**a**) and rate (**b**) of *Alternaria alternata* after 7 days of incubation. (C+: positive control, *Alternaria alternata* only). Different letters above the error bars indicate significant differences between treatments (*p* < 0.05) according to Duncan’s test, where treatments sharing the same letters are not significantly different. The value represents the mean of the 10 replicates ± standard error.

The mean mycelial growth inhibition was 44.6% at the lowest dose (C1) of *P. yamanorum*, while raising the concentration to 20% (C2) considerably increased the inhibitory action to 53.52%. Strong antifungal potential was exhibited by the maximum inhibition, which was observed at 40% (C3), where the fungal radial development was reduced by 64.32% (Figure 4a). Higher filtrate concentrations considerably inhibited fungal expansion, according to these results, which highlight a statistically significant dose–response relationship. The results of mycelial growth rate (mm/h) directly demonstrated this inhibitory trend (Figure 4b). Mycelial growth rate (MGR) retardation was noted as the *P. yamanorum* culture filtrate concentration increased. The average MGR values were 1.50 mm/h for C1, 1.35 mm/h for C2, and 1.18 mm/h for C3, compared to 2.16 mm/h of the positive control. The combined effect of increased MGI and retarded MGR as bacterial concentration increased illustrates its double-pronged effect on fungal growth dynamics.

These findings highlight the potential of *P. yamanorum* filtrates as a concentration-responsive biocontrol agent by indicating that increasing the dose significantly suppresses the development of *A. alternata*, both in terms of growth extent and speed.

### 2.4. Evaluation of Pseudomonas yamanorum Filtrate Dilutions on Tomato Seedling Growth

Tomato seeds were treated with different dilutions (D0, D10, D50, D100, D200, and D500) of the *P. yamanorum* filtrate to investigate the appropriate dilution for promoting seedling growth. Seven days following treatment, the length of the seedlings was measured and compared to the untreated control (C-).

The ANOVA results revealed a significant difference in seedling growth amongst the treatments (*p* < 0.05). The undiluted bacterial filtrate (D0) resulted in the highest mean seedling length, reaching up to 7.7 cm, which was significantly longer than the control group mean length of 4.4 cm. With slight variation from D0, the D10 also had a similar effect, illustrated by a mean seedling length of 6.7 cm, which suggests that the bioactivity of *P. yamanorum* was maintained even after a 10-fold dilution.

**Figure 5:**
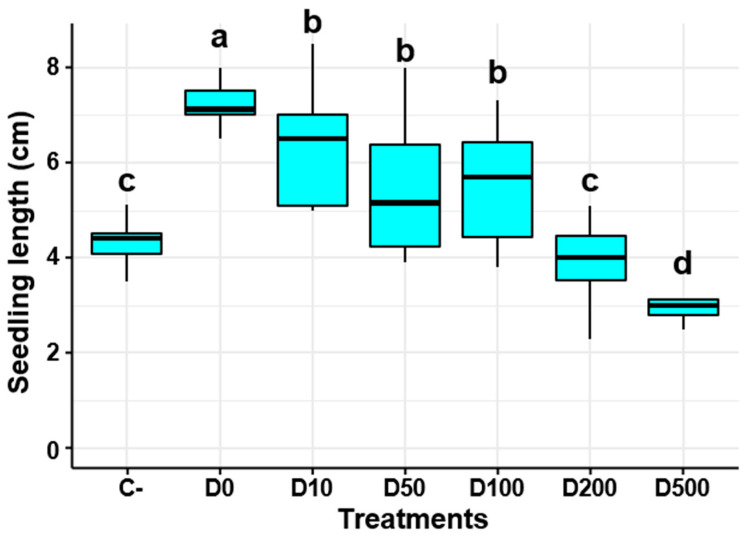
Effect of *Pseudomonas yamanorum* culture filtrate dilutions (D0, D10, D50, D100, D200, D500) on tomato seedling growth. C-: negative control (water only). Different letters above the boxplots indicate significant differences among treatments according to Duncan’s test (*p* < 0.05), where treatments sharing the same letter are not significantly different. The value represents the mean of the 30 replicates ± standard error.

The seedling growth-promoting effect at moderate dilutions, such as D50 and D100, was less than that of D0 and D10, indicating a dose-dependent response. Despite this fact, the growth-promoting effect remained higher than that of the negative control (C-). Moving toward a higher dilution of D200 and D500, the seedling length significantly declined (*p* < 0.01), with D500 displaying the lowest mean seedling length of 3.0 cm. According to these results, D0 and D10 are the best concentrations for promoting tomato seedling growth, with D0 exhibiting a stronger effect (Figure 5).

### 2.5. In Planta, Biocontrol Effect of Pseudomonas yamaranoum and Salicylic Acid Against Alternaria alternata and Plant Growth Promoting Efficacy Under Greenhouse Conditions

#### 2.5.1. Disease Severity

The efficacy of preventive treatments with *P. yamanorum* alone or combined with salicylic acid (SA) against *A. alternata* under greenhouse conditions is presented in Table 1.

At 30 dpi, all applied treatments (T1–T4) exhibited low disease severity scores ranging between 0.5 and 0.66, significantly (*p* < 0.01) lower than the positive control (T5) with a severity mean of 1.33.

By 60 dpi, disease symptoms increased in the positive control T5 (3.50), whereas the treatments maintained moderate disease inhibition. It was noted that T1 (10^7^ CFU/mL) resulted in the least severity score of 1.66, compared to T2 (10^8^ CFU/mL; 2.00), indicating that improved disease control is not necessarily linked to higher bacterial concentration. The T3 treatment (10^7^ CFU/mL + SA) exhibited a similar outcome (2.16), suggesting that SA at this point is producing significant synergistic effects. During the final assessment, disease severity peaked in T5 exhibited by the highest score of 6. Both T1 (2.5) and T3 (3.16) remained significantly more effective than T2 (3.33) and T4 (SA only; 4).

Furthermore, while SA alone (T4) exhibited a reduction in disease severity compared to T5, it was less effective than the bacterial treatments. These results demonstrate that *P. yamanorum* at 10^7^ CFU/mL provides optimal biocontrol efficacy against *A. alternata* under greenhouse conditions. On the other hand, the addition of SA may offer a slight enhancement but does not significantly outperform the bacterial treatment alone.

#### 2.5.2. Impact of *Pseudomonas yamanorum* and Salicylic Acid on Plant Growth Promoting Parameters

**Figure 6:**
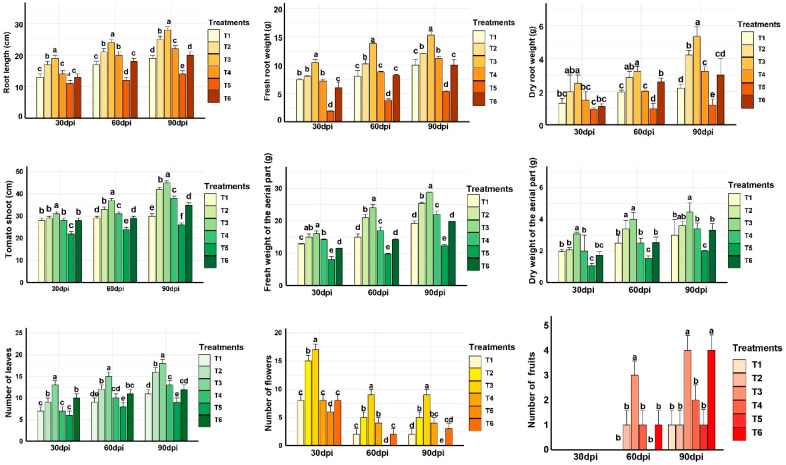
Growth promotion of tomato plants by *Pseudomonas yamanorum* and salicylic acid at three sampling moments (30, 60, and 90 days post inoculation). (T1: *P. yamanorum* 10^7^ CFU mL^−1^ + *A. alternata*), T2 (*P. yamanorum* 10^8^ CFU mL^−1^ + *A. alternata*), T3 (*P. yamanorum* 10^7^ CFU mL^−1^ + salicylic acid + *A. alternata*), T4 (salicylic acid + *A. alternata*), T5 (*A. alternata*), T6 (untreated). Different letters above the error bars indicate significant differences between treatments (*p* < 0.05) according to Duncan’s test, where treatments sharing the same letters are not significantly different. The value represents the mean of the 20 replicates ± standard error.

The application of *P. yamanorum* and salicylic acid (SA) demonstrated significant effects on various plant growth-promoting parameters in tomato plants, under *A. alternata* stress (Figure 6). The data collected at 30, 60, and 90 dpi enable comprehensive comparisons across treatments and may explain how the tomato plants’ response changed.

At 30 dpi, significant early differences were already noticeable (*p* < 0.01). The treatments T2 and T3 exhibited the most growth stimulation. This suggests that both treatments rapidly initiated mechanisms that boost tomato seedling vigor, potentially through early root colonization and induction of growth. While T1 showed moderate effects at early stages.

Advancing in time, 60 dpi, the preventative effect among treatments became more pronounced. T2 and T3 demonstrated the most optimal performance across all the investigated vegetative parameters. T3 reached the longest root length (24 cm), highest shoot length (37 cm), highest leaf number (15), and the heaviest aerial fresh weight (24 g), highlighting the benefits of *P. yamanorum* combined with salicylic acid. On the other hand, the pathogen-infected control T5 had only 8 leaves, 24 cm shoot length, and 12 cm root length, with minor values in all the other growth parameters. Salicylic acid alone (T4) had a modest effect, better than T5 but consistently lower than bacterial treatments. These results continued to confirm that the preventive inoculation of *P. yamanorum* combined with SA (T3) significantly enhances vegetative growth under biotic stress.

At the last assessment by 90 dpi, T3 continued to lead across most parameters: root length (28 cm), shoot length (45 cm), leaf number (18), and fresh weight of aerial part (29 g). T2 maintained strong performance as well with 25 cm root length, 42 cm shoot length, and 16 leaves. T1, which received a lower bacterial concentration, showed moderate performance. While T5 exhibited the lowest values across all the parameters. This confirms the cumulative growth-promoting effect of *P. yamanorum* treatments, mostly in T2 and T3.

For reproductive traits, fruit number at 90 dpi was highest in T3 (four fruits), comparable to T6, which is the healthy control (four fruits), whilst T4 had two fruits. T1 and T2 produced only one fruit, the same as T5 (one fruit). These findings suggest that SA enhances the reproductive effect of *P. yamanorum* when applied together (T3), perhaps by improving stress tolerance.

Overall, the temporal assessment of growth parameters from 30 to 90 dpi highlights that T3 (*P. yamanorum* + SA) offers the most accurate enhancement in both vegetative and reproductive performance. T2 (*P. yamanorum* at 10^8^ CFU/mL) is also highly effective for growth. These results demonstrate the impact of the bacterial strain *P. yamanorum as* a promising biocontrol and growth-promoting agent.

In addition to tomato growth parameters assessment, this study evaluates the chlorophyll content dynamics, which provide important insights into photosynthetic efficiency and plant vigor under biotic stress (Figure 6). At 7 dpi, all treatments displayed comparable chlorophyll levels (ranging from 35.46 to 38.33 SPAD), indicating a similar initial physiological status.

From 30 dpi, clearer distinctions emerged. T6 (healthy control) showed the highest chlorophyll content (53 SPAD); the other treatments showed similar values, with no effect, which suggests a possible stress-induced chlorophyll retention without functional gain.

**Figure 7:**
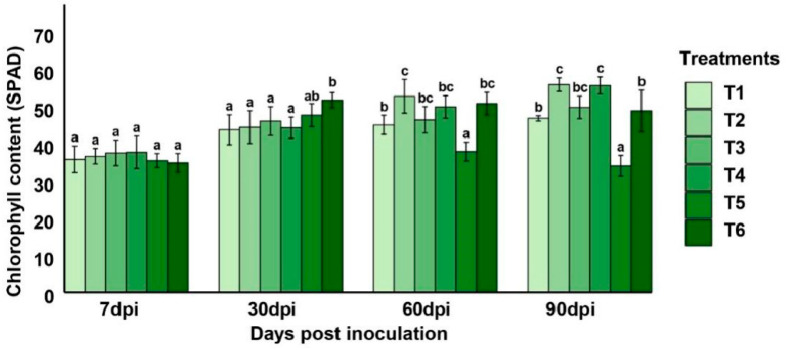
Impact of preventive treatments of *Pseudomonas yamanorum* and salicylic acid on the chlorophyll content in tomato leaves infected with *Alternaria alternata* at three sampling moments (30, 60, and 90 days post-inoculation). (T1: *P. yamanorum* 10^7^ CFU mL^−1^ + *A. alternata*), T2 (*P. yamanorum* 10^8^ CFU mL^−1^+ *A. alternata*), T3 (*P. yamanorum* 10^7^ CFU mL^−1^ + salicylic acid + *A. alternata*), T4 (salicylic acid+ *A. alternata*), T5 (*A. alternata*), T6 (untreated). Different letters above the error bars indicate significant differences between treatments (*p* < 0.05) according to Duncan’s test, where treatments sharing the same letters are not significantly different. The value represents the mean of the 20 replicates ± standard error.

After 60 days from treatment inoculation, it was observed that T2 (53.7 SPAD) and T4 (50.8) exceeded T3 (47.2), whereas T5 declined to 38.51, suggesting severe physiological impact from *A. alternata*. These results parallel vegetative growth trends: T2 and T3 showed the highest root/shoot lengths and biomass, while T5 had the least. At 90 dpi, T2 and T4 showed peak chlorophyll values of 56.9 and 56.6, respectively, in synchrony with higher shoot and root growth values (Figure 6). T3 also retained high chlorophyll content (50.63), while T5 declined drastically to 34.62, confirming disease progression. Overall, bacterial treatments with SA, T2, and T3 consistently boosted chlorophyll accumulation and translated this physiological advantage into measurable growth and reproductive success, which was still much higher in comparison to the positive control (T6; 49.68). T4, despite not matching T3 in shoot/root biomass, maintained high chlorophyll values (Figure 7).

#### 2.5.3. Enzymatic Activities

*Ascorbate peroxidase activity (APX)*

The combination of *P. yamanorum* (10^7^ CFU mL^−1^) and salicylic acid (T3) resulted in the highest ascorbate peroxidase activity response in tomato leaves, with values increased from 43.46 units mg^−1^ protein^−1^ min^−1^ at 7 dpi to 67.01 at 90 dpi, indicating a long-term effect. This may suggest a synergistic effect between *P. yamanorum*, salicylic acid, and the host’s defensive metabolism. Other treatments showed an increase in APX levels in the early stages from 7 dpi to 30 dpi, but declined significantly at 90 dpi. Similarly, T3 outperformed all other treatments in roots, maintaining the highest APX activity throughout the experiment. In this case, APX activity increased from 29.11 units.mg protein^−1^·min^−1^ at 7 dpi to 57.90 at 90 dpi, indicating systemic upregulation of antioxidant defense. Compared to the negative control (T6), it recorded an increase from 26.82 at 7 dpi to 50.54 at 90 dpi, reaching levels close to T3, while the positive control showed a decline, accounting for 9.74 at 90 dpi.

*Catalase activity (CAT)*

Over the 90-day post-inoculation period with *A. alternata*, the catalase (CAT) activity in tomato plants varied significantly (*p* < 0.01) according to treatment, indicating the oxidative stress response and effectiveness among the applied treatments. In leaf tissues, the highest increase was recorded in T3, where CAT activity increased from 37.78 at 7 dpi to 94.84 units mg^−1^ protein min^−1^ at 90 dpi, hence outperforming all the other treatments. This enhanced production suggests an active antioxidant response, most likely induced by the activation of the plant defense system. Moderate responses were observed in T1, T2, and T4 peaking at 53.48, 44.08, and 64.74 at 30 dpi, respectively, and then decreasing, suggesting overall weaker activation. In the roots, T3 compared to the positive control had the highest CAT activity, reaching 77.94 at 90 dpi, demonstrating its systemic effect on plant defense. These findings indicated that *P. yamanorum* treatments, particularly in combination with salicylic acid, may stimulate a more robust and long-lasting antioxidant response in both aerial and root tissues, increasing plant resistance to oxidative stress caused by *A. alternata.*

*Peroxidase activity*

**Figure 8:**
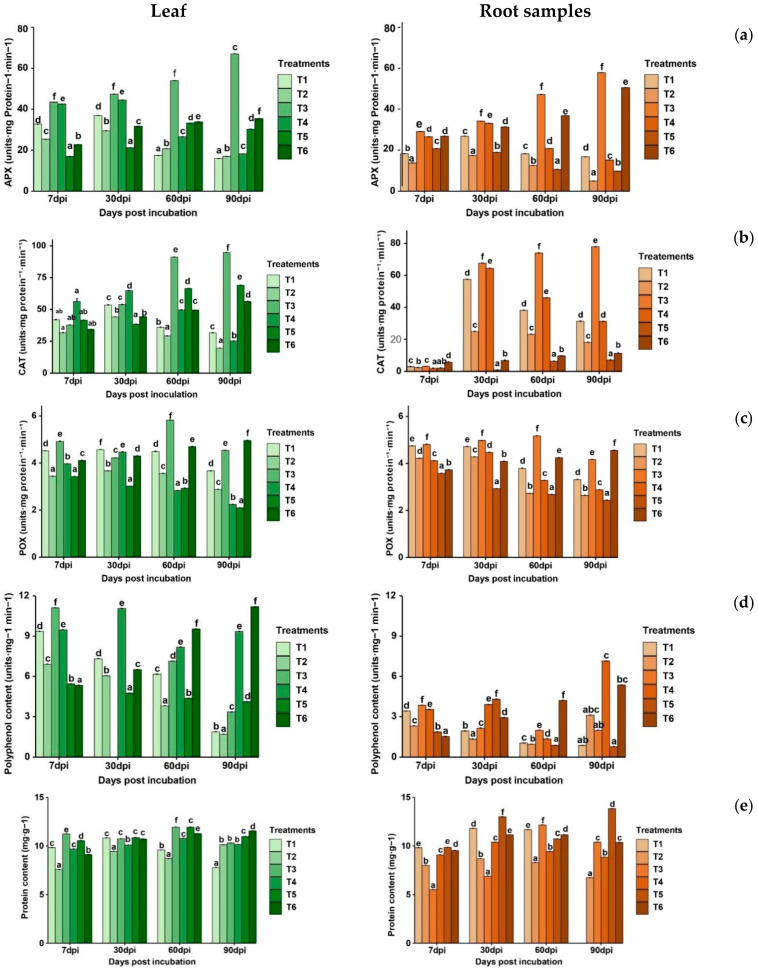
Impact of treatments on tomato plant defense mechanisms of tomato leaf and roots samples at four sampling moments (7, 30, 60, and 90 days post inoculation) (APX: Ascorbate peroxidase (**a**); CAT: Catalase (**b**); POX: peroxidase (**c**); Polyphenol content (**d**); protein content (**e**)). (T1: *P. yamanorum* 10^7^ CFU mL^−1^ + *A. alternata*), T2 (*P. yamanorum* 10^8^ CFU mL^−1^ + *A. alternata*), T3 (*P. yamanorum* 10^7^ CFU mL^−1^ + salicylic acid + *A. alternata*), T4 (salicylic acid + *A. alternata*), T5 (*A. alternata*), T6 (untreated). Different letters above the error bars indicate significant differences between treatments (*p* < 0.05) according to Duncan’s test, where treatments sharing the same letters are not significantly different. The value represents the mean of the 20 replicates ± standard error.

Peroxidase activity varied significantly (*p* < 0.01) across treatments in leaf and root tissues (Figure 8c). In tomato leaves, the combined treatment (T3) of *P. yamanorum* with salicylic acid exhibited the highest POX levels, peaking at 5.83 units mg^−1^ protein min^−1^ at 60 dpi, then slightly declining to 4.54 at 90 dpi. The negative control T6 maintained moderate POX levels, increasing from 4.11 at 7 dpi to 4.96 at 90 dpi. The POX levels observed in T6 remained close to those of T3 at 90 dpi, suggesting that the combined treatment was effective in enhancing plant defense responses to levels comparable to a healthy plant.

In root tissues, T3 also demonstrated the strongest POX activity across all sampling time points, reaching a maximum of 5.18 at 60 dpi and remaining comparatively high (4.17) at 90 dpi. On the other hand, the pathogen significantly impacted plant defense response, as indicated by the lowest POX activity recorded in T5, which declined from 3.58 at 7 dpi to 2.44 at 90 dpi. Bacterial effect was only presented by T1 and T2, both exhibited moderate activity in the early stages but declined notably by 90 dpi, suggesting minor durable responses.

*Polyphenol activity*

The combined treatment (T3) produced a significant and early accumulation of polyphenols, especially in leaves, which peaked at 12.09 units mg^−1^ protein min^−1^ at 30 dpi and then started to decline (Figure 8d) However, salicylic acid treatment showed a delayed yet sustained increase, especially at 90 dpi, where T4 recorded the highest polyphenol levels in both tissues (9.35 in leaves and 7.15 in roots). These trends suggest that while T3 promotes an early defense response, T4 may activate more durable systemic defense mechanisms. The positive control (T5) showed the lowest polyphenol levels, indicating a compromised defense response under pathogen stress.

*Protein content*

In response to preventive treatments, the total protein level in both leaves and roots in the presence of *A. alternata* infection varied significantly (*p* < 0.01). In the leaves, the treatment T3 (*P. yamanorum* 107 CFU mL^−1^ + salicylic acid) promoted higher protein accumulation, peaking at 60 dpi with 11.94 mg g^−1^ and then declining to 10.31 mg g^−1^ by 90 dpi. The positive control as well showed close protein levels matching with T3 at 60 dpi (11.94 mg g^−1^) and remaining high through 90 dpi (10.98 mg g^−1^), while the healthy control revealed the highest protein values at 90 dpi (11.56 mg g^−1^). T1 resulted in the lowest protein accumulation during all sampling periods, particularly in early stages, indicating a possible inefficiency in defense activation. On the other hand, in the roots, T3 initially exhibited low protein content (5.51 mg g^−1^ at 7 dpi), followed by an increase at 60 dpi (12.17 mg g^−1^). Despite the lowest levels exhibited by T1 in leaves, it supported better protein accumulation in roots, accounting for 15.82, which probably indicates not only a stress response but also an improved metabolic activity by *P. yamanorum*. The T1 may demonstrate that, in addition to reducing the effects of the disease, the biocontrol agent increased host metabolism, either by promoting growth or inducing systemic resistance. The positive control (T5) also showed sustainable protein levels over time compared to other treatments, with T5 reaching 13.84 mg g^−1^ and T1 peaking at 15.82 mg g^−1^ by 90 dpi. This may be explained as a stress-induced plant protective response to contain damage.

### 2.6. Effect of Pseudomonas yamanorum and Salicylic Acid on Tomato Fruit Quality

#### 2.6.1. Efficacy of Treatments for Postharvest Control of *Alternaria alternata* in Tomato Fruits

**Figure 9:**
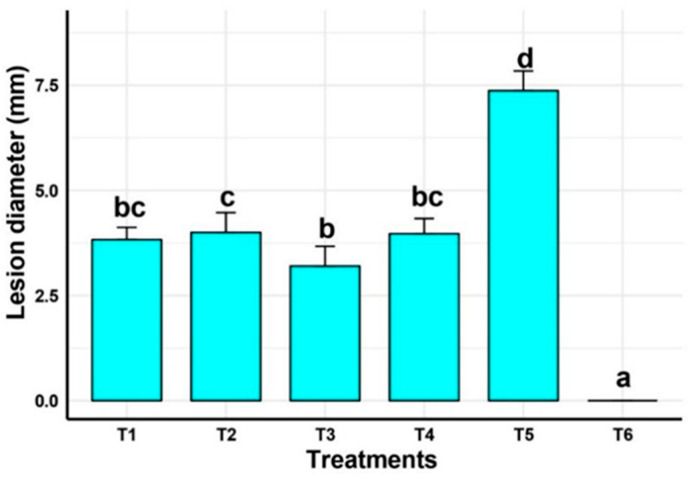
Efficacy of *Pseudomonas yamanorum* and salicylic acid in reducing lesion diameter caused by *Alternaria alternata* in Firenze tomato fruits. T1 (*P. yamanorum* 10^7^ CFU mL^−1^ + *A. alternata*), T2 (*P. yamanorum* 10^8^ CFU mL^−1^ + *A. alternata*), T3 (*P. yamanorum* 10^7^ CFU mL^−1^ + salicylic acid (C_6_H_4_(OH)CO_2_H) + *A. alternata*), T4 (salicylic acid + *A. alternata*), T5 (*A. alternata* only), T6 (untreated). Treatments marked with different letters indicate significant differences according to Duncan’s multiple range test (*p* < 0.05). Data are the average of 30 tomato fruits per treatment.

The effectiveness of *P. yamanorum* and salicylic acid treatments in preventing *Alternaria alternata*-induced lesion development in Firenze tomato fruits is demonstrated in Figure 9. In comparison to the infected control (T5), which displayed the largest lesion width of 7.37 mm, all biocontrol treatments significantly (*p* < 0.01) reduced lesion diameter. The untreated control (T6) exhibited no lesions, confirming the pathogenicity of *A. alternata* and the effectiveness of protective treatments.

The T3 treatment (*P. yamanorum* 10^7^ CFU/mL + salicylic acid) had the least lesion diameter (3.20 mm) among the infected fruits across the treatments, suggesting that the bacterial isolate and salicylic acid had a synergistic effect. With no statistical difference from T3, both T1 (*P. yamanorum* 10^7^ CFU/mL) and T4 (salicylic acid) also considerably decreased lesion size to 3.83 mm and 3.97 mm, respectively. This could suggest that T1 and T4 have the same impact, confirming T3’s effectiveness. The concentration of *P. yamanorum* 10^8^ CFU/mL showed a slightly larger lesion diameter (4 mm) than other treatments. This suggests that a slightly high concentration of the bacteria may stress the plant and adversely impact the efficiency of treatment. These findings generally indicate that salicylic acid and the biocontrol agent of *P. yamanorum* both have protective properties against *A. alternata* in postharvest conditions, with the combined treatment (T3) providing the best disease suppression.

#### 2.6.2. Effect of Preventive Treatments on Biochemical and Physiological Parameters of Fruit Quality

**Figure 10:**
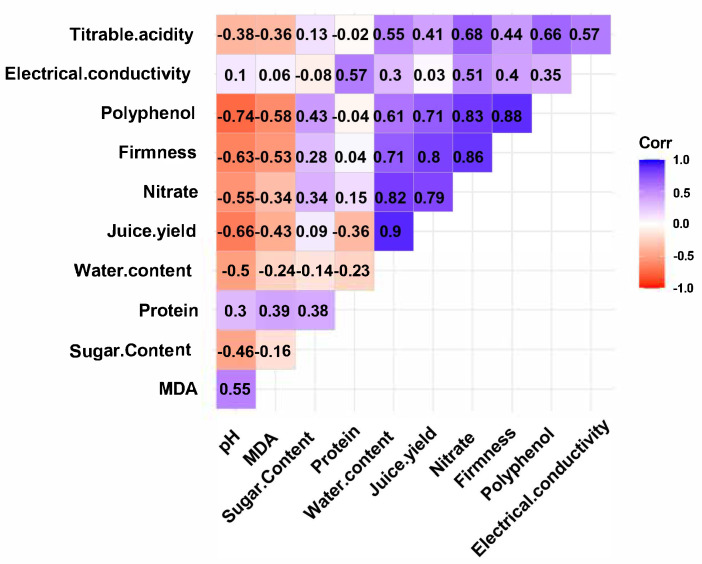
Pearson’s correlation coefficients (r) from the analysis of tomato fruit quality attributes, including pH, MDA, sugar content, protein, water content, juice yield, nitrate, firmness, polyphenol, and electrical conductivity.

The ANOVA test revealed a significant difference among treatments (*p* < 0.01), which indicated a high influence of the applied treatments on fruit quality (Table 2). The effect of the combination of *P. yamanorum* 10^7^ CFU/mL with salicylic acid showed an important overall performance, compared to other treatments and the healthy control. Fruit firmness was highest in T3, T4, and T6, with values ranging from 3.13 to 3.43, confirming that SA and *P. yamanorum* maintained fruit texture, while the positive control T5 displayed the lowest firmness (0.5). *P. yamanorum* of T1 produced the highest titratable acidity, 7.13, suggesting a better preservation of taste and postharvest quality. Salicylic acid applied in combination with *P. yamanorum* (T3) exhibited the lowest electrical conductivity (0.59 mS/cm), indicating better membrane integrity. Additionally, the combined treatment T3 significantly decreased the pH of the fruits (4.02) compared to the positive control (4.71), which matches that of healthy fruits (T6), which had a pH of 4.06. Sugar content was highest in T5 and T6 (6.53 and 7.5 °Brix), possibly due to stress-related sugar accumulation in T5; however, T3 (6.43 °Brix) also exhibited values close to the healthy control, which may indicate the efficiency of the combined treatment in maintaining sugar levels in fruits. Healthy fruits had the highest nitrate concentration, followed by T3 and T4, whilst T5 had the lowest, which may be a sign of poor nitrogen metabolism under pathogen stress. Water content and juice yield were most abundant in T3 and T6 (93% and 92%, respectively), showing their effectiveness in maintaining fruit hydration and processing quality. The MDA levels, reflecting oxidative stress, were higher in T2 (2.25 µmol/g), while relatively low in T3 (2.02), matched with healthy fruits (2.04), though this was associated with tissue damage rather than oxidative protection. Protein content peaked in T4 (3.22 mg/g) and T6 (3.57 mg/g), suggesting enhanced biosynthetic activity, furthermore, polyphenol content critical for antioxidant defense was highest in healthy fruits T6 (1.04 mg/g) and in combined treatment T3 (0.81 mg/g), but lowest in T2 (0.4) and the positive control T5 (0.39). Correlation analysis showed that polyphenol content was strongly positively correlated with firmness (r = 0.88), nitrate concentration (r = 0.83), and juice yield (r = 0.71), suggesting its role in maintaining fruit quality (Figure 9). Firmness was also highly correlated with nitrate concentration (r = 0.86) and juice yield (r = 0.80). However, MDA was negatively correlated with polyphenols (r = –0.58) and firmness (r = –0.53), indicating that lower oxidative stress is associated with better texture and antioxidant content. Overall, the biocontrol agent of *P. yamanorum* 10^7^ CFU/mL with salicylic acid provided synergistic effects, offering a sustainable strategy that maintained or improved tomato fruit quality under pathogen stress (Figure 10).

## 3. Discussion

In vitro experiments confirmed the salient antagonistic activity of *P. yamanorum*, with significant inhibition of fungal mycelial growth (up to 68.7% in dual culture assays) and spore germination (88.7%) at a concentration of 10^8^ CFU/mL. The culture filtrate as well showed a dose-dependent suppression of fungal growth and reduced mycelial growth rate. In seedling bioassays, the undiluted and 10-fold diluted bacterial filtrates significantly improved radicle and hypocotyl development, with the highest vigor index recorded at D0. Preventive treatments with *P. yamanorum*, particularly at 10^7^ CFU/mL combined with salicylic acid (SA), successfully reduced disease severity throughout the 90-day trial. The treatments T3 (10^7^ CFU/mL + SA) and T1 (10^7^ CFU/mL alone) were the most effective in curtailing disease severity. In terms of growth and plant development, these treatments also led to significant increases in root and shoot length, leaf number, and aerial biomass. In particular, T3 consistently led to improved vegetative and reproductive traits, including a higher number of fruits. The application of *P. yamanorum*, particularly when combined with SA, enhanced the antioxidant defense system of the tomato plants. Activities of catalase, peroxidase, and ascorbate peroxidase were significantly elevated in both leaves and roots, alongside higher total polyphenol and protein contents, indicating the induction of systemic resistance. Chlorophyll content also remained higher in treated plants, suggesting improved photosynthetic efficiency under biotic stress. These results support the efficacy of *P. yamanorum* as a promising biocontrol, which also promotes plant health and fruit quality in tomato cultivation under early blight stress. The findings of this study are in tandem with those of previous studies that have explored other *Pseudomonas* isolates, such as *P. aeruginosa* FG106, which produced hydrolytic enzymes and antifungal compounds inhibiting *A. alternata* and other plant diseases [22], and *P. fluorescens* RB5 secreted proteases, chitinases, and siderophores that disrupted fungal mycelia [23]. These observations indicate that *P. yamanorum* probably secretes non-volatile substances (phenazines, pyrrolnitrin, pyoluteorin, or similar antibiotics) and lytic enzymes (chitinase, protease, and lipase) to colonize *A. alternata*. It also confirmed the production of siderophores and HCN to compete for iron, thus exhibiting antifungal effects. In addition to antagonism, *P. yamanorum* exhibited classic plant-growth-promoting (PGP) attributes. Plant growth-promoting traits are well documented in several studies of *Pseudomonas* PGPR. The strain S3 isolated from turmeric rhizosphere synthesized around 37 µg mL^−1^ IAA, solubilized significant phosphate, produced siderophores, HCN, and ammonia, and exhibited antagonism towards *Rhizoctonia* disease [24]. Likewise, *P. aeruginosa* strains are able to produce IAA, siderophores, and HCN while solubilizing phosphate [22,25]. By enhancing root development and nutrient uptake, as well as stimulating plant defenses and suppressing diseases, such activities improve plant growth both directly and indirectly. In this study, under biotic stress, the potential of *P. yamanorum* in producing IAA and nutrient-solubilization abilities probably contributed towards the observed increases in root and shoot growth. Moreover, the destruction of fungal cell walls by *P. yamanorum* enzymatic activities, including cellulase, protease, and lipase, might further explain its strong in vitro suppression of *A. alternata* [23].

In accord with other studies, the studied bacterial inoculum with high concentrations of CFU 10^7^ to 10^8^ CFU/mL yielded optimal disease control, *B. velezensis,* as well as showing that around 10^6^ to 10^8^ CFU/mL is often required for effective rhizobacterial biocontrol of *A. solani* in the tomato crop [26]. On the other hand, salicylic acid (SA) and *P. yamanorum* together further enhanced protection, indicating a synergistic activation of plant defenses. In fact, PGPR can prime jasmonic acid and ethylene signaling pathways, and SA application is known to induce systemic acquired resistance (SAR) as well as defense gene expression [27].

The tomato plants responded to *P. yamanorum* with an increased defense mechanism, which was beneficial at the biochemical level. Compared to infected controls, treated plants exhibited significantly higher levels of antioxidant enzymes. These enhancements are hallmark signatures of induced systemic resistance (ISR) and stress mitigation. Similar patterns have been studied for other PGPR organisms, such as the *Bacillus* spp., which induce higher peroxidase, polyphenol oxidase, and superoxide dismutase activities in tomato, contributing to systemic resistance against early blight [28,29,30]. The increase in CAT, APX, and POX in our investigation demonstrates that *P. yamanorum* may contribute to the detoxification of reactive oxygen species (ROS) produced by *A. alternata* infection, thereby preserving cellular components and maintaining leaf health [31]. Our findings ascertain that *P. yamanorum* combined with salicylic acid (SA) reflects integrated strategies of microbial biocontrol with elicitor-induced systemic resistance. Regarding plant growth promotion, it was demonstrated that *Pseudomonas putida* and salicylic acid gave more significant results in terms of seedling vigor and enhanced agronomic parameters, dovetailed with the outcomes of our trials [32].

The results of this study are in congruence with preceding research on the application of biocontrol agents, such as *Bacillus* sp. and *Pseudomonas* sp., in controlling plant pathogens [20,33,34,35]. Investigating and selecting a novel local species (*P. yamanorum*) with potent capabilities is crucial in promoting plant growth and development whilst also competently suppressing disease-causing agents. Rather than frequently applying synthetic fungicides, which consequently lead to long-term environmental problems, public health issues, and pathogen resistance, *P. yamanorum* provides a paradigm shift towards sustainable disease control. This microbial treatment lowers early blight incidence and promotes plant growth, resulting in healthy postharvest fruits, whilst minimizing inputs like fungicides and chemical fertilizers, thus reducing post-harvest losses and enhancing the profitability of the tomato production enterprise [33,36].

## 4. Materials and Methods

### 4.1. Fungal Pathogen

The phytopathogen fungus, *A. alternata*, was isolated from tomato fruits showing symptoms of early blight disease and cultured on Potato Dextrose Agar (PDA) medium at 25 ± 2 °C. Macroscopic, microscopic, and molecular identification was performed to confirm the fungus species identity.

DNA extraction was performed using Quick-DNA Fungal/Bacterial MiniPrepTM Kit (Zymo Research Group, Tustin, CA, USA), following the manufacturer’s guide protocol. Polymerase chain reaction (PCR) was carried out to amplify the internal spacer region ITS using the universal primers ITS1 (5′-TCCGTAGGTGAACCTTGCGG-3’) and ITS4 (5’-TCC TCCGCTTATTGATATGC-3’). PCR cycling conditions were as follows: initial denaturation at 94 °C for 1 min, followed by 35 cycles of 94 °C for 30 s, 58 °C for 30 s, 72 °C for 1 min, and then a final extension at 72 °C for 10 min. All PCR products were separated by electrophoresis on a 1.5% agarose gel, stained with SYBR Safe DNA Gel Stain (Invitrogen, Carlsbad, CA, USA), and visualized under UV illumination. Three positive amplified PCR products were excised from the gel, then purified and sequenced by Sanger sequencing (Applied Biosystems, Bedford, MA, USA).

The obtained sequences were edited and quality checked by analyzing the chromatogram peaks using BioEdit 7.2.5 software [37]. The identity and similarity of the sequences were checked by Blast in the NCBI database [38] and then aligned and compared with reference sequences, using the MEGA V.7 software [39]. The sequences of the pathogen were then deposited in GenBank under accession number: PQ892125.

### 4.2. Bacterial Antagonist

The *P. yamanorum* used in this study was provided from the Plant Protection and Biological Sciences Laboratory at the Regional Centre for Agricultural Research (CRRA) in Sidi Bouzid, Tunisia (GenBank accession number: PQ555427, [20]).

### 4.3. Physiological Properties of P. yamanorum

Pure colonies of the bacterial strain were cultured on Luria–Bertani agar (LB) and incubated for 48 h, then aseptically scraped and transferred into 250 mL Erlenmeyer flasks each containing 250 mL of LB broth. The flask cultures were incubated at 28 °C in a rotary shaker incubator (Optic Ivymen System, Barcelona, Spain) at 150 rpm, and the bacterial concentration was adjusted to 10^8^ CFU/mL using a spectrophotometric measurement at 600 nm. This standardized bacterial inoculum was used for all subsequent assays and diluted according to the experimental protocol.

To evaluate the physiological properties of *P. yamanorum*, bacterial growth was assessed at different temperatures and pH levels. Growth in LB medium was tested at 15 °C, 30 °C, and 45 °C. For pH tolerance, the medium was adjusted to pH 4, 5, 7, 9, and 11 using HCl or KOH. Each parameter was tested with five replicates, and after 48 h of incubation under each set of conditions, visible growth was recorded as a positive indicator of bacterial viability.

### 4.4. Enzyme Production and Plant Growth Promotion Traits

The ability to produce extracellular enzymes was assessed for *P. yamanorum***,** including catalase (cat), amylase (amy), protease (pro), pectinase (pec), and glucanase (glu), using techniques employed by Hajji-Hedfi et al. [20]. Additionally, properties that are essential for plant growth-promoting activities were noted, such as Phosphate solubilization (P), Indole Acetic Acid (IAA) production, Hydrogen cyanide (HCN) production, and nitrogen fixation (N). All assays were performed in five replicates for each parameter, and assays were repeated three times to confirm the reproducibility of the results.

**Catalase assessment:** A volume of *P. yamanorum* culture was transferred from a Luria–Bertani (LB) medium into a glass slide, and then a drop of hydrogen peroxide (3%) was mixed with the samples. Positive catalase activity is indicated by the production of air bubbles.

**Amylase assessment:** Amylase activity was determined on Nutrient Agar (NA) supplemented with 1% (*w*/*v*) soluble starch. Plates were inoculated using a sterile inoculation loop, then incubated at 30 °C for 48 h. After visible growth appeared, the agar surface was flooded with an aqueous iodine solution and thereafter washed with distilled water. A clear halo surrounding the colony indicates starch hydrolysis, while a negative result, on the other hand, shows a brown tint surrounding the culture.

**Protease assessment:** NA medium supplemented with 5% skim milk was used to assess protease activity. Plates were spot inoculated with bacterial cultures and incubated for 4 days at 30 °C. Protease production was indicated by the appearance of a clear halo surrounding the bacterial colony, indicating casein hydrolysis. A negative result was recorded when no clearing zone formed around the growth colony.

**Pectinase assessment:** Pectinase production evaluation was carried out by inoculating the bacterial cultures on Pectin Congo Red agar, and then incubating at 30 °C for 24 h. Pectinase production was indicated by a clear halo forming around the bacterial growth.

**Glucanase assessment:** *P. yamanorum* was inoculated onto an agar medium supplemented with 1% barley flour as a source of β-1,3-glucans, then the plates were incubated at 30 °C for 5 days. Following incubation, the medium was flooded with a 0.1% (*w/v*) Congo red solution. The development of clear zones around the bacterial colonies was a sign of β-1,3-glucanase activity.

**Phosphate solubilization determination:** A volume of 10 µL of the bacterial suspension was inoculated on Pikovskaya’s agar supplemented with tri-calcium phosphate [40], then the plates were incubated at 30 °C for 7 days. A positive result for phosphate solubilization was indicated by clear halo zones formed around the bacterial colonies [41].

**Determination of IAA production:** An 80 mm diameter Wattman paper disk treated with Salkowski’s reagent (2% of FeCl3 at 0.5 M in 35% of Perchloric acid) was placed on the surface of bacterial inoculated LB medium supplemented with a concentration of 5 mM of Tryptophan, 0.06% of SDS, and 1% glycerol which was incubated at 28 °C for 48 h. The production of IAA was indicated by the formation of a pink color around inoculated bacteria after the addition of Salkowski reagent. Quantification of IAA production was determined by measuring absorbance at 530 nm using a spectrophotometer (Thermo Scientific Evolution 100, Thermo Fisher Scientific, Waltham, MA, USA), as described by Milagres et al. [42].

**Determination of Hydrogen cyanide (HCN) production.** HCN production by the studied bacterial strain was examined using the method described by Pranaw et al. [43]. The bacterial strain was inoculated on agar medium supplemented with 4.4 g L^−1^ of glycine. A 90 mm Whatman filter paper was saturated with 0.5% picric acid solution in 2% sodium carbonate and lined in the lid of the Petri dish. The plates were then sealed with Parafilm, incubated at 28 °C for 4 days, and monitored for color change. Hydrogen cyanide production was indicated by the change in color of the filter paper from yellow to orange-brown.

**Nitrogen fixation assessment:** The nitrogen fixation potential of the bacterial strain was assessed using nitrogen-free agar medium and incubated at 30 °C for 48 h. The presence of visible bacterial growth on the plates after incubation served as a positive indicator of the isolate’s ability to fix atmospheric nitrogen (N_2_) [41].

### 4.5. In Vitro, the Antagonistic Effect of Pseudomonas yamanorum Against Alternaria alternata

#### 4.5.1. Dual Culture Assays

Bacterial strain was evaluated for its potential antagonistic activity against *A. alternata*, using a dual culture assay culture growth method [44], with the standardized bacterial concentration. A 5 mm disc of *A. alternata* seven-day-old culture was placed onto one side of a 9 cm Petri dish of PDA medium (10 mL), and on the other side, 10 µL of bacterial strain (1.5 × 10^8^ CFU mL^−1^) was placed at 3 cm from the pathogen mycelium. The mycelial growth was monitored daily in mm for 7 days following Hajji-Hedfi et al. [21]. The inhibition percentage was calculated using the formula:

(1) $$\mathrm{Inhibition}\;\mathrm{percentage}\;(\%)=\frac{L-I}{L}\times 100$$

where L: the radial growth of fungi in control, and I: the radial growth of fungi in the presence of the tested bacterial strain.

Indirect confrontation is applied by assembling two plates, stacking them with the pathogenic agent on top and the antagonist agent at the bottom. The junction between the two plates was sealed with Parafilm to prevent any loss of volatile substances. The cultures were incubated for 7 days at 28 °C [45]. Both assays were repeated twice, and each treatment had five replicates.

#### 4.5.2. Bacterial Culture Filtrates Effect on Pathogen Mycelial Growth and Spore Germination

The antifungal activity of bacterial strain filtrates was evaluated on the mycelial growth inhibition of *A. alternata* using dual culture techniques, with different concentrations, which were used separately for their efficacy. The bacterial filtrates were prepared according to the method used by Hajji et al. [33]. A total of 25 mL of the bacterial filtrate, adjusted separately at the concentrations of 10% (C1), 20% (C2), and 40% (C3), was incorporated into the PDA medium. Then, a 0.6 cm diameter mycelial disc of the pathogenic fungus was placed at the center of the PDA of each plate. For each concentration, five plates were included, and the entire assay was repeated twice. The plates were incubated at 28 °C for 7 days, mycelial growth was measured, and the percentage of mycelial growth inhibition (MGI %) was calculated at the end of the incubation period using the following formula:

(2) MGI (%) = (1 − Ce/Ct) × 100

where Ce is the radial growth diameter of *A. alternata* in the presence of bacterial filtrate. Ct is the radial growth diameter of *A. alternata* in the absence of filtrate.

Additionally, the mycelial growth rate (MGR) was measured according to formula (3), as reported by [20].

(3) MGR (mm/h) = [D1/Te1] + [(D2 − D1)/Te2] + [(D3 − D2)/Te3] + … [(Dn − Dn − 1)/Ten]

The calculation considers the fungus’s radial growth diameter (D) over a seven-day period and the associated incubation time (Te). The MGR is calculated by dividing the corresponding incubation time intervals by the sum of the continuous diameter changes.

To assess the efficacy of bacterial filtrate in inhibiting fungal spore germination, 200 µL of the bacterial culture was mixed with 200 µL of the fungal spore suspension in sterile Eppendorf tubes and supplemented with 1 mL of sterile distilled water containing 5% glucose and incubated for 24 h at 25 °C. Control tubes were inoculated only with the spore suspension of the selected pathogen.

Germination inhibition (GI %) was calculated using the following formula:

(4) $$\mathrm{GI}\;\%=\frac{SGc-SGt}{SGc}\times 100$$

where *GSc*: number of germinated spores in the control tubes, *SGt*: number of germinated spores of the pathogen in the presence of the antagonist. A hemocytometer was used to adjust the spore counts, and after incubation, the number of germinated spores was determined microscopically using a Malassez cell [20].

### 4.6. Evaluation of Biocontrol Potential of P. yamanorum on Tomato Seed and Seedling

Tomato seeds cv. Firenze were sterilized with ethanol and then rinsed twice with sterile distilled water before drying at room temperature. After that, the seeds were treated separately with different bacterial dilutions of 10, 50, 100, 200, and 500 times, for 30 min. The treated seeds were placed on paper towels moistened with sterile distilled water and kept in aerated sterile plastic bags at 30 °C for germination. For each dilution, 10 replicates were performed per treatment, and the assay was repeated three times to ensure data reliability. After 7 days, hypocotyl and radicle (seedling) lengths were measured to calculate the vigor index (VI) of the seedlings as per the formula:

(5) VI = Percentage of germination × (length of the hypocotyl + length of the radicle)

### 4.7. In Planta Evaluation of P. yamanorum and Salicylic Acid Treatments on Tomato Plants for A. alternata Control

#### 4.7.1. Disease Severity Assessment

To evaluate the effect of the bacterial isolate, *P. yamanorum*, and salicylic acid under greenhouse conditions as preventive treatments against *A. alternata*, experiments were conducted at the CRRA, Sidi Bouzid, Tunisia, in 2022–2023, using tomato plants (cv. Firenze) provided by a certified nursery. The plants were cultivated in plastic pots filled with a substrate composed of one-third peat, one-third sterile sand, and one-third sterile soil. The treatments were applied as follows: T1 (*P. yamanorum* 10^7^ CFU mL^−1^ + *A. alternata*), T2 (*P. yamanorum* 10^8^ CFU mL^−1^ + *A. alternata*), T3 (*P. yamanorum* 10^7^ CFU mL^−1^ + salicylic acid (C_6_H_4_(OH)CO_2_H, 0.001 M) + *A. alternata*), T4 (salicylic acid+ *A. alternata*), T5 (*A. alternata* only), T6 (untreated). *P. yamanorum* and salicylic acid (10 µL each) were applied first, and then, 24 h later, *A. alternata* spore suspension (10 µL of conidial suspension 10^6^ spores/mL) was inoculated. To evaluate disease response, ten replicate plants per treatment were employed in a randomized complete block design, and the entire experiment was repeated twice. The plants were then maintained in a greenhouse at 25 °C for a period of 90 days to monitor disease development and other aspects of physiological responses, as well as agronomic growth parameters, as mentioned above.

Disease severity was monitored at 30, 60, and 90 days post-inoculation (dpi), based on a disease severity scale. Symptoms of early blight were scored using a scale from 0 to 6, according to Hajji-Hedfi et al. [33]. Scores on this scale corresponded with the extent of leaf surface covered by lesions: 0 (no lesions), 1 (1–5% leaf surface), 2 (6–10% leaf surface), 3 (11–20% leaf surface), 4 (21–35% leaf surface), 5 (36–50% leaf surface), and 6 (51–100% leaf surface).

#### 4.7.2. Physiological Responses and Agronomic Growth Parameters

Chlorophyll content was measured using a portable fluorimeter (OS1p; NH 03051, USA), and the results were expressed in SPAD units. Measurements were recorded at 7, 30, 60, and 90 dpi. The morphometric parameters as agronomic growth indicator were recorded at 30, 60, and 90 dpi, including root length (cm), fresh root weight (g), shoot height (cm), fresh weight of the aerial part (g), dry weight of the aerial part (g), number of leaves, number of flowers and number of fruits.

#### 4.7.3. Antioxidant Enzymatic Activities

To investigate the biochemical responses of tomato plants to preventive treatments against *A. alternata*, the enzymatic activities were measured in root and leaf tissues. Measured enzymes were ascorbate peroxidase (APX), catalase (CAT), peroxidase (POX), Polyphenol content, and Protein content. Samples of root and leaf from each treatment were taken at 7, 30, 60, and 90 dpi, then were immediately frozen in liquid nitrogen to avoid enzyme degradation.

For extraction, one gram of fresh root and leaf was homogenized in 5 mL of extraction buffer (1 mM PMSF, 0.2 mM EDTA, 1% PVP in 50 mM phosphate buffer, pH 7.0) using a mortar and pestle . The homogenate was centrifuged at 12,000× *g* for 10 min at 4 °C, and the resulting supernatant was collected and stored at −20 °C for enzyme analysis.

Peroxidase (POX) activity was measured following the method described by Reddy et al. [46]. Catalase (CAT) activity was measured at 240 nm, following the method of Hajji-Hedfi et al. [21]. Ascorbate peroxidase (APX) activity was determined at 290 nm, according to the method established by Singleton and Rossi [47]. The protein content was determined according to Bradford’s method [48], and polyphenolic content was measured using the Folin–Ciocalteu reagent, as described by Singleton and Rossi [47].

### 4.8. Fruit Quality Analysis

#### 4.8.1. Efficacy of Post-Harvest Pathogenicity Assay

The tomato fruits were harvested from the greenhouse in the 2022–2023 agricultural season. They were surface-sterilized with 1% sodium hypochlorite, rinsed with sterile distilled water, and surface-dried at room temperature. Selected tomatoes of uniform size were inoculated with 20 µL of each treatment (T1 to T6) via injection. After incubation for 2 h at ambient temperature, 20 µL suspension of spores of the pathogen (*A. alternata*) was inoculated into the same wound site to evaluate the efficiency of the treatments. Ten treated fruits were then individually placed in sterile Petri dishes containing cotton pads moistened with 10 mL of sterile distilled water to maintain high humidity. The lesion diameter (mm) caused by rot development was measured for the seven days of incubation. The entire assay was replicated three times for data reliability.

To assess the antifungal efficiency of the applied treatments on tomato fruits, the pathological, morphometric, physicochemical, and biochemical characteristics were examined after seven days, as detailed below.

#### 4.8.2. Fruit Quality Assessment

Fruit quality was assessed by analyzing several parameters. Fruit firmness was determined according to guidelines by Geasa and Hassan [49]. Furthermore, physical-chemical parameters such as water content, juice yield, pH, titratable acidity, sugar content, nitrate content, and electrical conductivity were assessed in the tomatoes.

Water content (WC) was determined according to the formula below:

(6) WC (%) = (FF − FS)/(FF) × 100

where FF is the fresh weight of the fruit and FS is the dry weight of the fruit.

The juice yield was determined by using the formula:

(7) JY (%) = (weight of juice)/(fresh weight of fruit) × 100

where JY is the juice yield, FF is the fresh weight of the fruit, and FS is the dry weight of the fruit.

For pH recording, the electrode of the pH meter was inserted into the tomato juice, thus measuring the pH. A digital pocket refractometer (Atago PAL, Tokyo, Japan) was used to measure the sugar content in degrees Brix. Using a LAQUA-twin nitrate meter (Horiba Ltd., Kyoto, Japan), the nitrate concentration was measured. Electrical conductivity (EC) was determined using a HI99301 conductivity meter (Hanna Instruments, Woonsocket, RI, USA). The titratable acidity (TA) of the tomato juice was determined according to Hajji-Hedfi et al. [21] and expressed in (g/10 mL Juice). Protein and polyphenol content were assessed in tomato fruits, as described above.

The level of lipid peroxidation was assessed by measuring malondialdehyde (MDA) content according to the method described by Haraguchi et al. [50], with slight modifications. Briefly, 200 mg of the homogenized tomato sample was mixed with 2 mL thiobarbituric acid (TBA), trichloroacetic acid (TCA), and hydrochloric acid (HCl). The mixture was then exposed to heat at 95 °C for 30 min. After that, the samples were immediately cooled on ice for 10 min and centrifuged at 12,000× *g* for 15 min to separate the supernatant. The absorbance of the supernatant was measured at 532 nm and 600 nm using a spectrophotometer. The MDA concentration was calculated using an extinction coefficient of 155 mM^−1^·cm^−1^, and results were expressed as µmol of MDA per gram of fresh matter (µmol/g).

### 4.9. Statistical Analysis

All statistical analyses were performed using R software version 4.5.1; data are expressed as mean ± standard error (SE). The data were checked for normality according to the Shapiro–Wilk test, and homogeneity of variances using Levene’s test. Analysis of variance (ANOVA) followed by Duncan’s multiple range test (DMRT) was applied to assess significant differences between treatment means at *p* ≤ 0.05. Diagram visualizations were created using the “ggplot2” package in RStudio (Version 2025.05.1+513).

## 5. Conclusions

This study demonstrated the potential of *P. yamanorum* as a biological control agent against *A. alternata* in tomato. Both in vitro and greenhouse trials confirmed its efficacy in reducing pathogen growth and disease severity. The investigated tomato plants showed enhanced physiological and biochemical responses, including elevated antioxidant enzyme activities, increased protein content, improved chlorophyll levels, and growth performance. The combined application of *P. yamanorum* and salicylic acid further amplified these effects due to their synergistic interaction that enhances disease resistance and promotes plant vigor. Further studies on the identification of compounds secreted from *P. yamanorum* to better understand the mechanism of interaction between the biocontrol agent and the pathogen are recommended. Alternatively, additional trials on dose selection are required to formulate an accurate bio-stimulant.

## Figures and Tables

**Table 1:** Effect of preventive treatments of *Pseudomonas yamanorum* and salicylic acid (SA) on the disease severity in tomato plants infected with *Alternaria alternata* at 30, 60, and 90 days post-inoculation.

Treatments	30 dpi	60 dpi	90 dpi
T1	0.66 ± 0.51 ab	1.66 ± 0.51 b	2.5 ± 0.54 b
T2	0.66 ± 0.51 ab	2 ± 0.63 b	3.33 ± 0.18 bc
T3	0.5 ± 0.4 ab	2.16 ± 0.40 b	3.16 ± 2.61 bc
T4	0.66 ± 0.51 ab	2.33 ± 0.51 b	4 ± 1.09 c
T5	1.33 ± 0.51 b	3.5 ± 1.22 c	6 ± 0.89 d
T6	0 ± 0 a	0 ± 0 a	0 ± 0 a
*p*-value	*p* < 0.01	*p* < 0.01	*p* < 0.01

ANOVA analysis. Means of a column followed by the same letter are not significantly different according to the Duncan test at *p* < 0.05 and *p* < 0.01. The value represents the mean of the 20 replicates ± standard error.

**Table 2:** Effects of *Pseudomonas yamanorum* and salicylic acid treatments on physiological and biochemical parameters of tomato fruits under *Alternaria alternata* stress.

Treatments	Firmness	pH	EC (mS/cm)	TA (g/10 mL Juice)	Sugar Content (°Brix)	Nitrate (mg/L)	WC (%)	Juice Yield (%)	MDA (µmol/ g DW)	Protein (mg/g)	Polyphenol
T1	2.17 ± 0.25 b	4.44 ± 0.04 ab	4.54 ± 0.36 c	7.13 ± 0.15 a	5.17 ± 0.75 d	1366.67 ± 152.75 c	92.01 ± 1 ab	40.34 ± 0.29 c	2.02 ± 0.02 b	1.6 ± 0.33 c	0.7 ± 0.08 c
T2	1.23 ± 0.15 c	4.84 ± 0.05 a	3.73 ± 0.05 d	5.73 ± 0.21 b	5.77 ± 0.15 c	1366.67 ± 57.74 c	91.34 ± 0.29 bc	35.44 ± 0.51 e	2.25 ± 0.06 a	3.22 ± 0.32 ab	0.4 ± 0.04 d
T3	3.13 ± 0.25 a	4.02 ± 0.04 b	0.59 ± 0.03 f	5.37 ± 0.55 b	6.43 ± 0.15 b	1566.67 ± 115.47 b	93.06 ± 0.08 a	55.88 ± 0.21 a	2.02 ± 0.04 b	1.47 ± 0.26 c	0.81 ± 0.04 b
T4	3.13 ± 0.25 a	4.64 ± 0.26 a	5.95 ± 0.07 a	5.33 ± 0.06 b	5.83 ± 0.12 c	1500 ± 100 bc	90.34 ± 0.09 c	37.05 ± 1 d	2.12 ± 0.04 b	3.58 ± 0.35 a	0.71 ± 0.03 c
T5	0.5 ± 0.17 d	4.71 ± 0.48 a	1.4 ± 0.11 e	4.7 ± 0.2 c	6.53 ± 0.06 b	690 ± 20 d	83.3 ± 1.54 d	16.99 ± 0.02 f	2.13 ± 0.04 ab	2.79 ± 0.16 b	0.39 ± 0.03 d
T6	3.43 ± 0.47 a	4.06 ± 0.04 b	5.48 ± 0.1 b	7.53 ± 0.25 a	7.5 ± 0.1 a	1966.67 ± 57.74 a	91.9 ± 0.17 ab	44.7 ± 0.2 b	2.04 ± 0.15 b	3.57 ± 0.45 a	1.04 ± 0.06 a
*p*-value						<0.01					

ANOVA analysis. Means of a column followed by the same letter are not significantly different according to the Duncan test (*p* < 0.05 and *p* < 0.01). (T1 *P. yamanorum* 10^7^ CFU mL^−1^+ *A. alternata*), T2 (*P. yamanorum* 10^8^ CFU mL^−1^+ *A. alternata*), T3 (*P. yamanorum* 10^7^ CFU mL^−1^ + salicylic acid + *A. alternata*), T4 (salicylic acid+ *A. alternata*), T5 (*A. alternata*), T6 (untreated). Data are the average of 30 tomato fruits per treatment.

## Data Availability

The original contributions presented in this study are included in the article. Further inquiries can be directed to the corresponding authors.
